# Delta radiomics for rectal cancer response prediction with hybrid 0.35 T magnetic resonance-guided radiotherapy (MRgRT): a hypothesis-generating study for an innovative personalized medicine approach

**DOI:** 10.1007/s11547-018-0951-y

**Published:** 2018-10-29

**Authors:** Luca Boldrini, Davide Cusumano, Giuditta Chiloiro, Calogero Casà, Carlotta Masciocchi, Jacopo Lenkowicz, Francesco Cellini, Nicola Dinapoli, Luigi Azario, Stefania Teodoli, Maria Antonietta Gambacorta, Marco De Spirito, Vincenzo Valentini

**Affiliations:** 1 Dipartimento di Diagnostica per Immagini, Radioterapia Oncologica ed Ematologia, Istituto di Radiologia, Fondazione Policlinico A. Gemelli IRCCS - Università Cattolica Sacro Cuore, Largo A. Gemelli, 8, 00168 Rome, Italy; 2 Dipartimento di Diagnostica per Immagini, Radioterapia Oncologica ed Ematologia, Fondazione Policlinico Universitario A. Gemelli IRCCS, Rome, Italy; 3 Dipartimento di Diagnostica per Immagini, Radioterapia Oncologica ed Ematologia, Istituto di Fisica, Fondazione Policlinico A. Gemelli IRCCS - Università Cattolica Sacro Cuore, Rome, Italy

**Keywords:** Rectal cancer, Radiomics, Delta radiomics, Personalized medicine, Innovative biotechnology, MRIdian, ViewRay

## Abstract

The aim of this study was to evaluate the variation of radiomics features, defined as “delta radiomics”, in patients undergoing neoadjuvant radiochemotherapy (RCT) for rectal cancer treated with hybrid magnetic resonance (MR)-guided radiotherapy (MRgRT). The delta radiomics features were then correlated with clinical complete response (cCR) outcome, to investigate their predictive power. A total of 16 patients were enrolled, and 5 patients (31%) showed cCR at restaging examinations. *T*2*/*T*1 MR images acquired with a hybrid 0.35 T MRgRT unit were considered for this analysis. An imaging acquisition protocol of 6 MR scans per patient was performed: the first MR was acquired at first simulation (*t*0) and the remaining ones at fractions 5, 10, 15, 20 and 25. Radiomics features were extracted from the gross tumour volume (GTV), and each feature was correlated with the corresponding delivered dose. The variations of each feature during treatment were quantified, and the ratio between the values calculated at different dose levels and the one extracted at *t*0 was calculated too. The Wilcoxon–Mann–Whitney test was performed to identify the features whose variation can be predictive of cCR, assessed with a MR acquired 6 weeks after RCT and digital examination. The most predictive feature ratios in cCR prediction were the L_least and glnu ones, calculated at the second week of treatment (22 Gy) with a *p* value = 0.001. Delta radiomics approach showed promising results and the quantitative analysis of images throughout MRgRT treatment can successfully predict cCR offering an innovative personalized medicine approach to rectal cancer treatment.

## Introduction

Significant improvements in locally advanced rectal cancer (LARC) treatment have been met in the past two decades, and to date the typical therapeutic workflow is represented by neoadjuvant long-course radiochemotherapy (RCT), followed by total mesorectal excision (TME) [1–4].

Regardless of the initial disease stage, approximately 11–42% of these patients achieve a pathological complete response (pCR) after long-course RCT. Different studies have shown that patients achieving pCR usually have a better prognosis in terms of local control (LC), metastases-free survival (MFS) and overall survival (OS) [5, 6].

Conservative surgical approaches have recently been investigated in patients showing clinical complete response (cCR) after neoadjuvant treatments: both local excision (LE) and “watch and wait” (W&W) approaches represent to date feasible options in order to reduce morbidities and toxicities related to unnecessary TME procedures [7–9].

In the framework of a fully personalized medicine, the possibility to predict the patients who will achieve cCR before surgery or even during neoadjuvant RCT is of crucial importance and the contribution of imaging techniques to this end is significantly increasing [10–12].

The still ambiguous correlation between cCR and pCR has been evaluated by Hiotis and colleagues, who demonstrated a cCR rate of 19% in a dataset of nearly 500 patients with a pCR rate of 25% among complete responders at restaging, while Smith et al. observed that 61.3% of patients with a pCR had evidence of incomplete clinical response at restaging [13, 14]. In a very recent systematic review and pooled analysis, however, patients undergoing W&W approach showed a 3-year overall survival of 93.5% (against a 90.1% rate for patients with pCR) and a non-regrowth free survival rate was of 89.2% at 3 years, supporting the favourable prognostic value of cCR already supposed by the first conservative experiences of Habr-Gama et al. [15, 16].

Several prediction models have been developed to predict pCR in LARC, providing clinicians with valuable decisional support systems (DSS) for multidisciplinary oncological care tailoring, so that patients “predicted as not responding” will take advantage of intensified treatments, while those “predicted as responding” will undergo a therapeutic approach more oriented to organ preservation [13].

A few studies suggest models for pCR prediction in LARC founded on clinical data information or on features extracted from previously acquired diagnostic images (i.e. staging scans) or during RCT, but the evidence about cCR prediction is still poor. The extraction of these significant features from biomedical images takes advantage of innovative biotechnologies, such as the radiomics analysis techniques that aim to a quantitative analysis of the images [17–19].

Cusumano et al. recently described a radiomics-based predictive model able to identify patients achieving pCR through the use of staging magnetic resonance (MR) images, while other authors investigated the possibility to identify such patients analysing the tumour volume on one or more MR scans acquired throughout the radiotherapy treatment [20–22].

The use of radiomics features variation in different imaging techniques throughout the treatment, defined as “delta radiomics”, has been proposed in the literature as a predictive tool for several oncological diseases, for both response and toxicity prediction outcomes [23–26].

Magnetic resonance imaging (MRI) appears to be a promising imaging technique for this application, due to its consolidated use for the specific purpose, the high level of soft-tissue contrast and the valuable anatomical detail in the definition of LARC response, but very scarce evidence about radiomics applications on low-tesla images is to date available [27].

The recently released MRIdian^®^ system (ViewRay Inc., Cleveland, OH, USA) joins a 0.35 T whole body MRI scanner and a radiation therapy delivery system composed of three Cobalt-60 sources (Tri-60-Co) or a 6MV linear accelerator.

This hybrid solution represents a great opportunity as it allows an MRI acquisition before each daily fraction of radiotherapy and assures high-quality visualization of the volume and movements of the tumour and of the surrounding organs at risk in the framework of real-time MRI-based adaptive radiotherapy [28].

The aim of this hypothesis-generating study is to investigate the feasibility of radiomics analysis of low-tesla hybrid MR images and to evaluate the possibility to correlate delta radiomics data with cCR in patients (pts) affected by LARC and undergoing neoadjuvant RCT [29].

The variation of the parameters extracted from such images has been correlated with cCR in order to investigate the prognostic value of this innovative approach.

## Materials and methods

### Patients selection criteria

Patients affected by locally advanced (cT2-4 and/or cN0-1, cM0) rectal adenocarcinoma, undergoing long-course neoadjuvant RCT with a low-Tesla tri-Co-60 MRI-Hybrid system have been enrolled for this study.

Specific informed consent and MRI safety screening forms were administered to all eligible patients.

Patients denying specific consent to MRgRT, presenting clinical contraindications to MRI (e.g. the presence of non-MRI-compatible implanted cardiac devices, claustrophobia or major psychiatric disorders) or younger than 18 years, were considered not suitable for this study.

### Treatment workflow and response assessment

Neoadjuvant long-course RCT was prescribed to the selected patients, according to a Simultaneous Integrated Boost 2 (SIB2) delivery protocol.

5500 cGy in fractions of 220 cGy were prescribed to planning target volume (PTV) 1, while 4500 cGy in fractions of 180 cGy to PTV2.

A total number of 25 fractions were therefore scheduled.

PTV1 was considered as tumour and correspondent mesorectum with a 7-mm isotropic margin; PTV2 as mesorectum in toto and selected lymphatic drainage stations according to disease stage with a 7-mm isotropic margin [30].

Concomitant chemotherapy with Capecitabine chronomodulate (1650 mg/mq)/5-Fluorouracil (5-FU) c.i. or an intensification schedule with Capecitabine (1300 mg/mq) plus Oxaliplatin (60 mg/mq) was prescribed, in relation to clinical stage and general conditions of the single patient.

Patients were immobilized in the supine position, using the Fluxboard device (Fluxboard^TM^, MacroMedics, The Netherlands) in an appropriate, fully personalized and comfortable configuration.

Clinical restaging was assessed 6–8 weeks after the end of RCT by digital rectal examination (DRE) and restaging MRI, according to our institutional guidelines and clinical practice.

In case of major or complete clinical response at restaging imaging, endoscopic examination was performed with random biopsies to confirm absence of disease.

Complete clinical response was defined by the presence of all these criteria, independently reviewed by the multidisciplinary tumour board members:

A. Complete absence of palpable masses at DRE
B. Restaging MRI findings:
  No lymph nodes detected or lymph nodes with short axis < 5 mm.
  No primary tumour residual at morphological and diffusion weighted imaging (DWI) series with complete integrity of rectal wall layers.
  Hypointense parietal thickening in *T*2 sequences without evidence of hyperintense residual lesions in DWI sequences or hypointense lesions in apparent diffusion coefficient (ADC) map.
C. No detection of residual lesions or the presence of a flat scar at endoscopic examination.

### Image analysis

An imaging protocol consisting in 6 MR acquisitions was applied to all patients.

The first MR scan was acquired during the treatment simulation procedures (*t*_0 Gy_), and the others were performed one every five fractions (*t*_11 Gy_, *t*_22 Gy_, *t*_33 Gy_, *t*_44 Gy_ and *t*_55 Gy_).

**Fig. 1:**
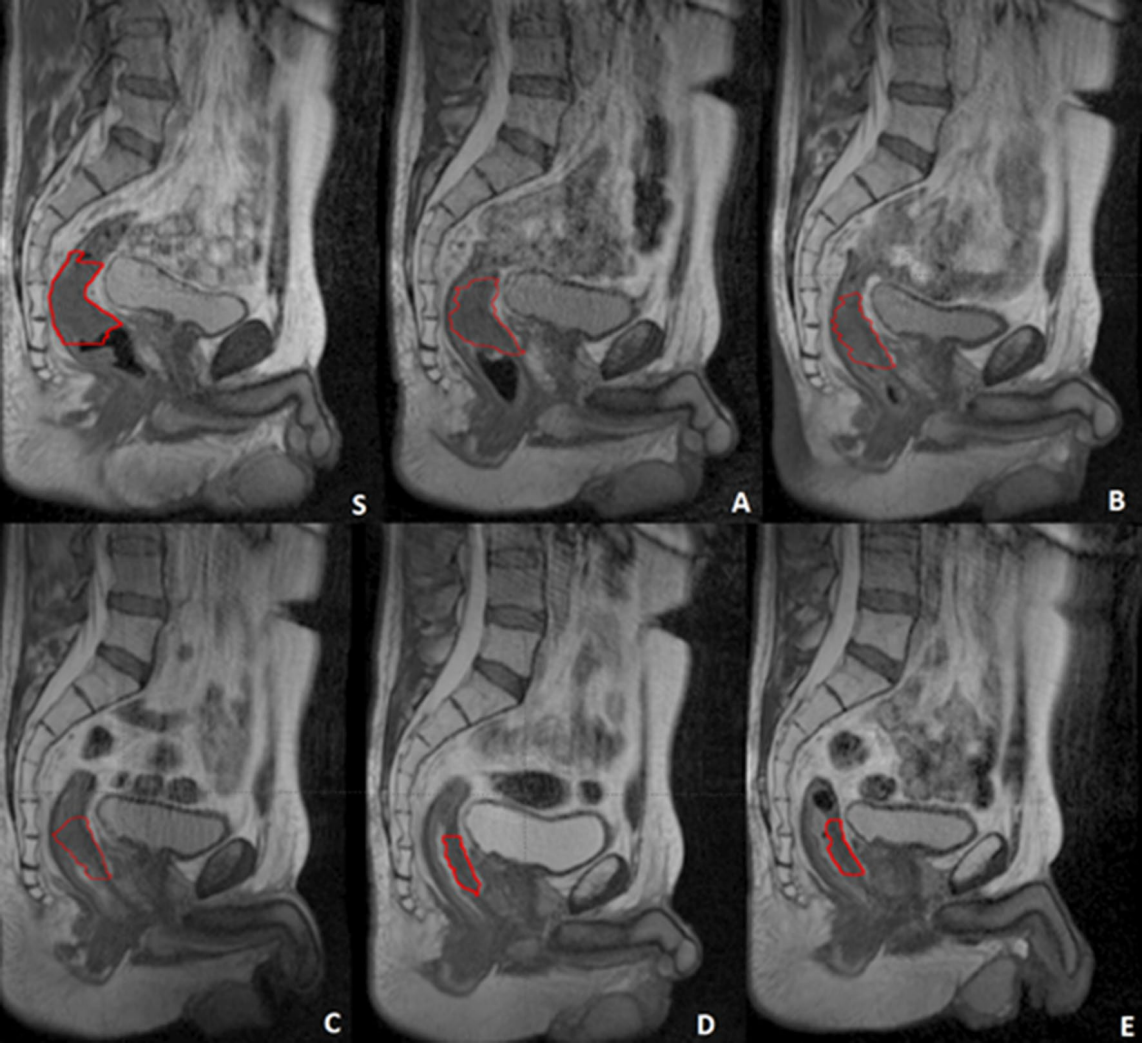
The complete image set of a patient from first simulation acquisition (*t*_0 Gy_) (S) to last fraction (*t*_55 Gy_) (**a**–**e**). The GTV is represented by the red contour

Figure 1 shows the imaging protocol applied in a case where a clinical complete response was achieved. The red contour indicates the GTV delineation.

All images were acquired on MRIdian using a TRUe Fast Imaging (TRUFI) with steady-state precession sequence, with image resolution of 1.5 × 1.5 × 1.5 mm^3^ and acquisition time of 175 s.

GTV was contoured slice by slice for all the image sets (*t*_0 Gy_–*t*_55 Gy_) by two radiation oncologists expert in the management of lower gastrointestinal malignancies.

The contoured images were then exported to Moddicom, a R library developed in our institution to perform radiomics analysis [31, 32].

The variation of the radiomics features that occurred during the treatment was quantified considering “delta features”.

Delta features were defined as the ratio between the features extracted from the images acquired at different treatment fractions (*t*_11 Gy_, *t*_22 Gy_, *t*_33 Gy_, *t*_44 Gy_ and *t*_55 Gy_) and the corresponding ones extracted from the image simulation (*t*_0 Gy_).

The Wilcoxon–Mann–Whitney (WMW) test was then performed to identify the features showing a predictive ability in discriminating patients undergoing cCR from those for which residual disease was expected [33].

Features showing a *p* value lower than 0.05 were considered statistically significant.

## Results

Sixteen consecutive patients (13 males and 3 females) affected by LARC (stages IIA–IIIC), with a median age of 64 years (range 49–86) were retrospectively enrolled for this study.

Five patients (31%) showed cCR at restaging examinations (DRE and MRI).

Patients characteristics are described in Table 1.

**Table 1:** Patients characteristics

Age	Sex	Site	CT	Stage	Restaging	Watch and wait
49	1	2	2	cT3 cN2 cM0	ycT0 ycN0 ycM0	1
80	1	1	1	cT4 cN2 cM0	ycT2/3 ycN1 ycM0	0
65	2	3	2	cT4 cN2 cM0	ycT0 ycN0 ycM0	1
75	1	2	1	cT4a cN1 cM0	ycT2 ycN0 ycM0	0
56	1	2	2	cT3 cN2 cM0	ycT2/3 ycN1 ycM0	0
77	1	3	1	cT4 cN0 cM0	ycT0 ycN0 ycM0	1
86	1	1	0	cT3 cN1 cM0	ycT0 ycN0 ycM0	1
61	2	2	1	cT3 cN0 cM0	ycT3 ycN0 ycM0	0
71	1	2	2	cT4a cN2 cM0	ycT0 ycN0 ycM0	1
62	1	2	2	cT3 cN2 cM0	ycT3 ycN1 ycM0	0
54	1	3	2	cT3 cN1 cM0	ycT2 ycN0 ycM0	0
69	1	2	1	cT3 cN1 cM0	ycT3 ycN0 ycM0	0
60	2	2	2	cT4 cN2 cM0	ycT4 ycN0 ycM0	0
55	1	2	2	cT3 cN1 cM0	ycT3 ycN0 ycM0	0
52	1	2	1	cT2 cN1 cM0	ycT2 ycN0 ycM0	0
54	1	2	2	cT3 cN1 cM0	ycT3 ycN0 ycM0	0

*Sex* 1 male, 2 female, *Site* 1 high, 2 medium, 3 low, *CT* chemotherapy: 0 no CT, 1 Capecitabine alone, 2 Capecitabine and Oxaliplatin, *Watch and Wait* 1 yes, 0 no

Median time interval between end of neoadjuvant RCT and restaging examinations was 62 days.

A total of 53 radiomics features belonging to four families (morphological, statistical, fractal and textural based on run-length matrix) were extracted from each raw image, without applying any image filter.

A total of 318 radiomics features were therefore obtained (53 features extracted from the simulation image and 265 calculated as delta features).

Of these, a total of 6 simulation features and 57 “delta radiomics” features showed a *p* value < 0.05 (WMW test) in discriminating cCR patients from the non-responding ones. Table 2 reports the features that are significant with the corresponding time of acquisition and *p* values.

**Table 2:** Significant statistical, morphological, fractal and textural features with the corresponding MR acquisition treatment fraction

*Feature type*	*t*_0 Gy_	*t*_11 Gy_	*t*_22 Gy_	*t*_33 Gy_	*t*_44 Gy_	*t*_55 Gy_
(S) Min	–	0.009	0.025	–	0.024	–
(S) Range	–	–	–	–	0.038	0.019
(S) Energy	–	0.025	0.002	0.009	0.006	0.028
(M) Surface	–	–	–	–	0.003	0.019
(M) Volume	–	0.049	0.003	–	0.006	0.028
(M) Areavolume	–	–	0.003	0.013	0.006	–
(M) L major	–	–	–	–	0.028	–
(M) L least	–	0.037	**0.001**	0.006	0.002	0.013
*(M) Compactness 1*	0.038	–	–	–	–	–
*(M) Compactness 2*	0.038	–	–	–	–	–
*(M) Sphdispr*	0.038	–	–	–	–	–
*(M) Sphericity*	0.038	–	–	–	–	–
*(M) Asphericity*	0.038	–	–	–	–	–
(F) MedianFD	–	0.038	–	–	–	–
(F) MinFD	–	–	0.013	–	–	–
*(T) glnu*	0.027	0.038	**0.001**	0.013	0.003	0.038
(T) sre	–	0.019	0.019	0.028	–	–
(T) lre	–	0.019	0.019	0.038	–	–
(T) hgre	–	–	0.038	–	0.013	0.009
(T) srhge	–	–	0.038	–	0.013	0.009
(T) lrhge	–	–	0.038	–	0.013	0.009
(T) rlnu	–	–	0.028	–	0.013	–
(T) rlnu norm	–	–	0.019	0.028	–	–
(T) rperc	–	0.019	0.019	0.028	–	–
(T) rlvar	–	–	0.019	0.038	0.018	–

Features obtained from the analysis of simulation images (*t*_0 Gy_) are reported in italic style. *p* values ≤ 0.001 are highlighted in bold

As reported in the tables, most of the delta radiomics features show a higher statistical significance in discriminating between cCR and not-cCR patients when compared to those extracted from the analysis of the simulation images.

More specifically, three delta features (energy, grey level non-uniformity and least axis length) showed a statistically significant association (*p* value < 0.05) with the considered outcome throughout the entire treatment course.

The most significant *p* values were observed for the L_least and glnu features (*p* value = 0.001), when their variation between the simulation imaging and the second treatment week *t*_22 Gy_ MR was analysed.

The technical characteristics of the statistically significant features are reported in Appendix.

**Fig. 2:**
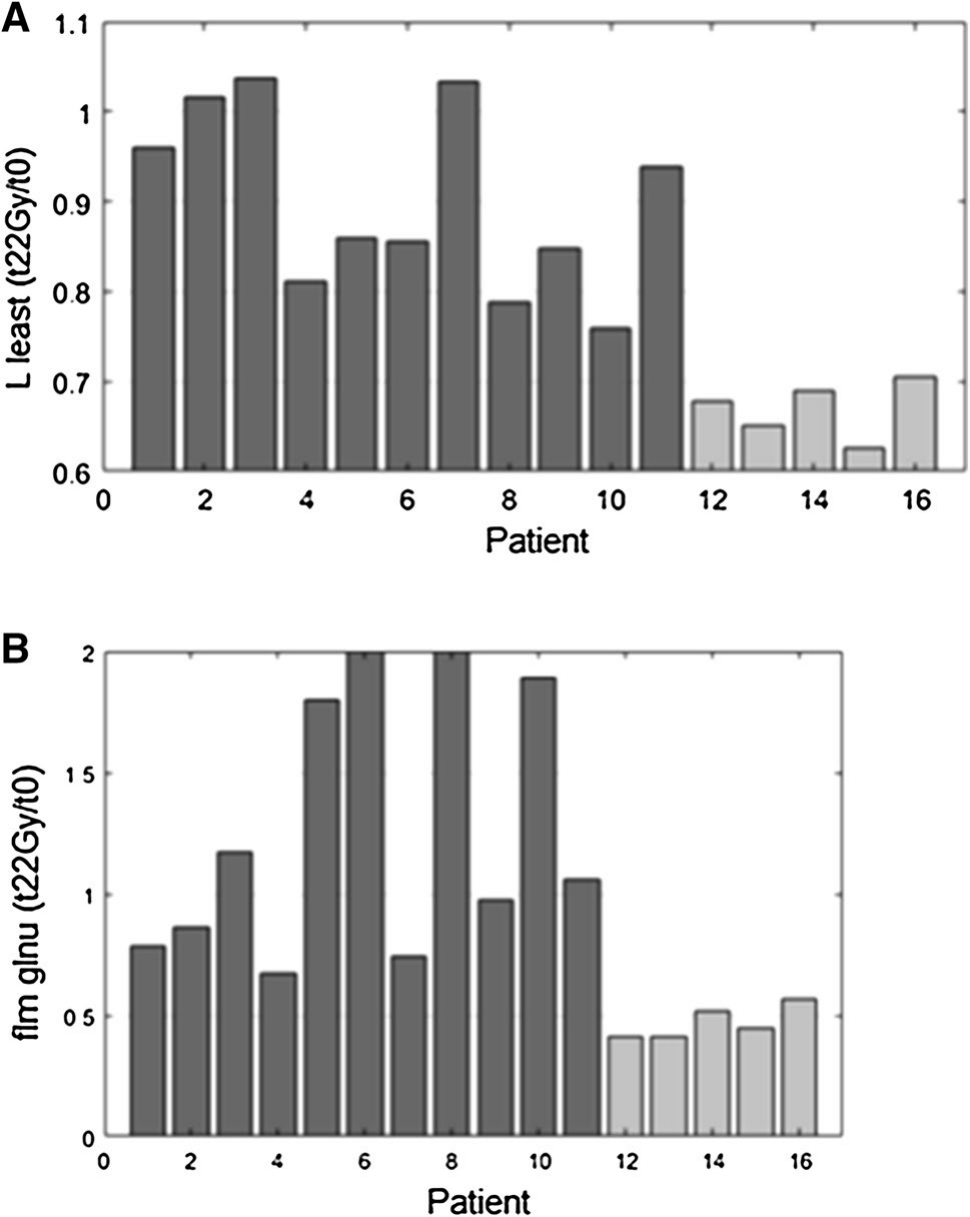
L least (**a**) and glnu (**b**) features trend. Patients undergoing cCR are indicated in light grey

Figure 2 shows the values of the two most significant aforementioned features for all the enrolled patients.

## Discussion

Imaging-based response prediction represents a very contemporary topic in rectal cancer management, allowing multimodal treatment tailoring and offering an important prognostic value for patient stratification, in the frame of the most modern personalized medicine approaches.

The usual workflow of radiomics-based radiotherapy studies generally considers imaging acquired in standard staging procedures (i.e. at least 1.5 T staging MRI) and assumes that the prescribed radiotherapy dose will be effectively delivered to the patient, without taking advantage of any information coming from IGRT imaging protocols during treatment delivery and limiting the models learning to a single image set [20, 34].

The very limited access to images acquired during treatment for radiomics purposes can be related to different causes, such as the present impossibility to extract reliable radiomics features from pelvic tumours CBCT, the extra dose to which the patient is exposed and the reduced access to diagnostic MRI scanners for studies [35].

This significant limit has been recently overcome by the availability of MRgRT daily setup images that allow the development of imaging-based response predictors during RCT treatment’s course with no additional burden in terms of dose to the patient, costs and procedures.

Indeed, the aim of this hypothesis-generating study was to explore the potentialities of the delta radiomics approach, more than to train a multiparametric prediction model, as the limited number of patients could not gather reliable conclusions.

The choice of using the Wilcoxon–Mann–Whitney test to investigate the predictive value of the delta radiomics parameters was adopted considering a recent study carried out by Parmar et al., who compared 14 different feature selection methods. Analysing the performance of these methods in different radiomics studies, the authors concluded that the WMW test is the most reliable and accurate method for feature selection in radiomics [36].

The obtained results suggest that delta features better discriminate between cCR and not-cCR patients reaching a statistical significance even higher of one order of magnitude when compared to the standard radiomics analysis performed on simulation imaging.

This increase in predictive power suggests that taking into account features variation during the course of the treatment, in addition to their absolute values at its beginning, could represent a significant added value for reliable outcome prediction.

Furthermore, the delta radiomics approach has the advantage of providing information related to the treatment course and the progressive response to multimodal therapies, allowing a real personalization of the treatment before its own end. The introduction of the described radiomics workflow can enrich the observations already available in the literature that took into account the predictive power of different tumour-related parameters during treatment: as an example, Palmisano et al. recently presented their experience about the impact of tumour volume reduction, evaluated with a mid-treatment diagnostic MRI scan, in the prediction of complete pathological response (pCR) in patients affected by rectal cancer [21].

Similar conclusions were reached also by van den Begin et al., through the use of serial diagnostic MRI [22].

Our results about tumour response are in general accordance with those obtained by these colleagues with different techniques, as hybrid MRgRT images showed the most promising radiomics applications for response prediction starting from the second week of treatment (*t*_22 Gy_).

The fact that several features coming from the textural analysis of low-tesla MR images show high significance in discriminating between cCR and not-cCR patients suggests that low-tesla MR images, although having an inferior signal to noise (SNR) ratio in comparison to diagnostic high-tesla MR images, could provide clinically valuable information.

Furthermore, Wachowicz et al. recently demonstrated that low magnetic field strength performs as well or better than higher fields in terms of contrast-to-noise (CNR) ratio of the images [37].

For these reasons, low-tesla MR images show inferior resolution with respect to diagnostic images but present a comparable or even better contrast.

As far as the authors know, this study represents the first radiomics evaluation of images acquired during the course of hybrid MRgRT in rectal cancer, introducing an innovative approach in the management of this disease.

Prospective validation studies based on this preliminary results and new related hypothesis-generating experiences are needed to confirm the clinical usability and the impact of these observations and to train “delta Radiomics” predictive models in order to improve and better define the optimal acquisition timing (i.e. taking into account all fractions or only the most significant ones).

## Conclusions

Our hypothesis-generating experience suggests that low-tesla MRgRT images can be suitable for radiomics analyses and opens new frontiers for the development of imaging-based prediction models.

Larger cohorts of patients are, however, needed to confirm these preliminary observations and to allow more reliable technical assumptions on this innovative radiomics workflow.

These applications may enhance the role of low-tesla hybrid MRgRT approach in rectal cancer management, theoretically reducing unnecessary overtreatment-related toxicities through treatment tailoring (e.g. surgery sparing in responding patients) and avoiding any increase in patient irradiation (e.g. CBCT-based radiomics) or diagnostic MRI scanner workload, saving time for patients waiting for disease staging and resources.

Our experience demonstrates that delta radiomics and imaging features variation during MRgRT treatment may represent a cCR prediction tool in locally advanced rectal cancer RCT and can be considered hint for new observations.

This innovative biotechnological approach adds promising resources to current personalized medicine in rectal cancer care and encourages treatment imaging radiomics analysis throughout the therapeutic workflow.

## Appendix

Technical characteristics of the statistically significant features are listed in Table 2.

All the definitions refer to the “Image biomarker standardisation initiative” by Zwanenburg et al. available at https://arxiv.org/abs/1612.07003.

### Statistical

- *Min* is represented by the lowest grey level present in the ROI.
- *Range* describes the statistical values of the grey levels range in the ROI.
- *Energy* indicates how grey levels are distributed inside the ROI.

### Morphological

- *Surface* represents the surface calculated summing over the triangular face surface areas of the ROI mesh.
- *Volume* represents the volume calculated from the ROI.
- *Areavolume* “Surface-to-volume ratio”. Ratio between surface and volume of the ROI.
- *L major* “Major axis length” determines the main orientation of the ROI, according to a principal component analysis (PCA) approach.
- *L least* “Least axis length” describes the axis along which the object is least extended according to a principal component analysis (PCA) approach.
- *Compactness 1* measures how compact, or sphere-like the volume is.
- *Compactness 2* measures how sphere-like the volume is.
- *Sphdispr* “Spherical disproportion” measures how sphere-like the volume is.
- *Sphericity* measures how sphere-like the volume is.
- *Asphericity* describes how much the ROI deviates from a perfect sphere.

### Fractal dimension

- *MedianFD* median value of all fractal dimensions calculated on the ROI slice by slice.
- *MinFD* minimum value of all fractal dimensions, calculated on the ROI slice by slice.

### Textural

- *glnu* “Grey level non-uniformity” describes the distribution of neighbouring grey level dependence counts over the grey values themselves.
- *sre* “Short runs emphasis” emphasizes short run length.
- *lre* “Long runs emphasis” emphasizes long run length.
- *hgre* “Low grey level run emphasis” emphasizes low grey levels.
- *srhge* “Short run high grey level emphasis” emphasizes runs in the lower left quadrant of the GLRLM.
- *lrhge* “Long run high grey level emphasis” emphasizes runs in the lower right quadrant of the GLRLM.
- *rlnu* “Run length non-uniformity” describes the distribution of runs over the run lengths.
- *rlnu norm* “Normalized run length non-uniformity”. Normalized version of rlnu.
- *rperc* “Run percentage” defines the fraction of the number of realized runs and the maximum number of potential runs.
- rlvar: “Run length variance” estimates the variance in runs for run lengths.
